# Insulin-Like Growth Factor 1 (IGF-1) Mediates the Effects of Enriched Environment (EE) on Visual Cortical Development

**DOI:** 10.1371/journal.pone.0000475

**Published:** 2007-05-30

**Authors:** Francesca Ciucci, Elena Putignano, Laura Baroncelli, Silvia Landi, Nicoletta Berardi, Lamberto Maffei

**Affiliations:** 1 Scuola Normale Superiore, Pisa, Italy; 2 Department of Psychology, Florence University, Florence, Italy; 3 Institute of Neuroscience CNR, Pisa, Italy; University of Maryland, United States of America

## Abstract

Enriched environment (EE) has been recently shown to affect visual cortex development and plasticity, and to prevent dark rearing effects. The factors mediating EE effects on visual cortical development and plasticity are still unclear. We have investigated whether IGF-1 is involved in mediating EE effects on the developing visual cortex. We show that EE increases the number of IGF-1 positive neurons in the visual cortex at P18. Increasing IGF-1 in the visual cortex of non-EE rats by means of osmotic minipumps implanted at P18 mimics EE effects, accelerating visual acuity development, assessed with Visual Evoked Potentials (VEPs). Blocking IGF-1 action in the visual cortex of EE rats by means of the IGF-1 receptor antagonist JB1 from P18 completely blocks EE action on visual acuity development. These results show that IGF-1 is a key factor mediating EE effects on visual cortical development. We then show that IGF-1 affects GAD65 immunoreactivity in perisomatic innervation and the condensation of Chondroitin Sulphate Proteoglycans (CSPGs) in perineuronal nets (PNNs) in the visual cortex. This suggests that IGF-1 action in mediating EE effects could be exerted through the modulation of intracortical inhibitory circuitry and PNN development.

## Introduction

Classic works [1], [2] show that physiology, biochemistry and morphology of the nervous system are affected by environmental enrichment (EE), a complex sensory-motor stimulation, used as experimental paradigm to test the influence of experience on the brain. EE affects the brain both at functional level, enhancing cognitive functions, particularly learning and memory [3]–[6] and at an anatomical level, promoting structural changes such as increment in hippocampal neurogenesis [7], [8], dendritic arborization [9] and synaptic density in cerebral cortex, hippocampus and cerebellum [10], [4].

EE has been recently shown to strongly affect visual cortex development and plasticity [11] and to prevent dark rearing effects on visual acuity development and critical period closure [12]. EE animals show an acceleration of visual acuity development, an increment of GAD65/67 protein at P7 and P15 and a precocious peak of BDNF expression at P7 [11].

Molecular mediators of anatomical and physiological changes observed in enriched animals are not yet completely understood. EE increases, in the adult, the production of several factors including neurotrophins, like NGF and BDNF [13], [14] strongly involved in visual cortical development and plasticity [15], [16].

A particularly good candidate as a mediator of EE effects is insulin-like growth factor 1 (IGF-1). Classically, IGF-1 has been implicated in prenatal and postnatal events in central nervous system development such as the control of cell proliferation, gliogenesis, neurogenesis, neuron survival, differentiation, synaptogenesis, myelination [17]–[20]. Recently, IGF-1 has been shown to mediate in the adult both the neuroprotective effects of physical exercise and possibly EE on neuronal death [21], [22], [23] and the enhancement caused by exercise in hippocampal plasticity and in learning and memory [24], [25], [20], [26]. Running induces uptake of IGF-1 by specific groups of neurons enhancing electrical activity [23] and increases IGF-1 expression in the hippocampus [26]; interestingly, EE has been shown to up-regulate IGF-1 receptor gene in the adult rat hippocampus and sensorimotor cortex [27].

A recent work [28] demonstrated that monocular deprivation (MD) increases the expression of IGF-1 binding protein and affects several genes in the IGF-1 pathway; exogenous application of IGF-1 prevents the physiological effect of MD on ocular dominance plasticity examined in vivo. This suggests that IGF-1 could be involved in experience-dependent plasticity in the visual cortex.

We have investigated whether IGF-1 is involved in mediating the effects of the experience provided by EE on postnatal visual cortical development using visual acuity development, a sensitive index of visual cortical development and predictive of behaviour [29], [30], [11]. We have already shown that EE strongly accelerates visual acuity development [11], [31]; EE mice and rats reach adult values of visual acuity already at postnatal day 25 (P25).

IGF-1 expression in the brain corresponds to regions and periods of axon outgrowth, dendritic maturation and synaptogenesis [32], [33]. Some studies [32], [34], [35] demonstrated that IGF-1 peak of expression is restricted to different time windows in different regions, according to the time course of their maturation. Nothing is known on the developmental profile of IGF-1 expression in the postnatal visual cortex. We have first assessed whether exposure to EE affects IGF-1 levels in the visual cortex between P15 and P25; this period, which follows eye opening, marks the beginning of the experience dependency of visual cortical development and the opening of the critical period for ocular dominance plasticity. We have found that at P18 the number of IGF-1 positive neurons is higher in the visual cortex of EE rats with respect to controls.

In a second experiment, we have increased IGF-1 levels in the visual cortex from P18 to P25 in non-EE rats infusing IGF-1 by means of osmotic minipumps and assessed whether this mimicked EE effects on visual acuity development. We found that IGF-1 infusion mimics EE effects on visual acuity.

In the reverse experiment we blocked IGF-1 action in the visual cortex of EE rats from P18 to P25 infusing the IGF-1 receptor antagonist JB1 and assessed whether this blocked EE action on visual acuity development. We found that the effects of EE were completely blocked.

To understand the possible mechanisms of action of IGF-1, we infused IGF-1 in the visual cortex of non-EE rats from P18 to P25 and assessed the developmental status of two key factors of visual cortical development and plasticity, the intracortical inhibitory circuitry and the CSPG containing PNNs. Development of intracortical inhibitory circuitry is well correlated with the increase in visual acuity [29], [36], [37] and the accompanying decrease in receptive field size of neurons in the primary visual cortex [38]. Precocious development of intracortical inhibition in BDNF overexpressing mice is accompanied by an accelerated development of visual acuity [29] and EE, which accelerates visual acuity development, also affects development of GABA biosynthetic enzymes [11]. CSPGs are component of the extracellular matrix recently shown to be non permissive for visual cortical plasticity [39], [40]. Their developmental organization into PNNs is well correlated with the decline of visual cortical plasticity accompanying the closure of the critical period for ocular dominance plasticity [39], [41] and EE, which prevents dark rearing effects on critical period, also prevents dark rearing effects on the development of CSPG containing PNNs [12].

We found that IGF-1 affected both intracortical inhibition and PNN development.

We conclude that IGF-1 is a key factor mediating EE effects on visual cortical development and is involved in experience-dependent plasticity in the developing visual cortex; its action could be mediated by an effect of intracortical inhibition and PNN development.

## Results

### EE affects the developmental time course of IGF-1 labelling in the visual cortex

Several works analysed IGF-1 mRNA expression and protein levels in the central nervous system during development [42], [32]; however, no data are available on IGF-1 presence in the developing visual cortex. We have therefore assessed IGF-1 levels in the visual cortex between P15 and P25 and then evaluated EE effects on this developmental time course. The period P15-P25, which follows eye opening, marks the beginning of the experience dependency of rat visual cortical development and the opening of the critical period for ocular dominance plasticity [38], [15].

**Figure 1:**
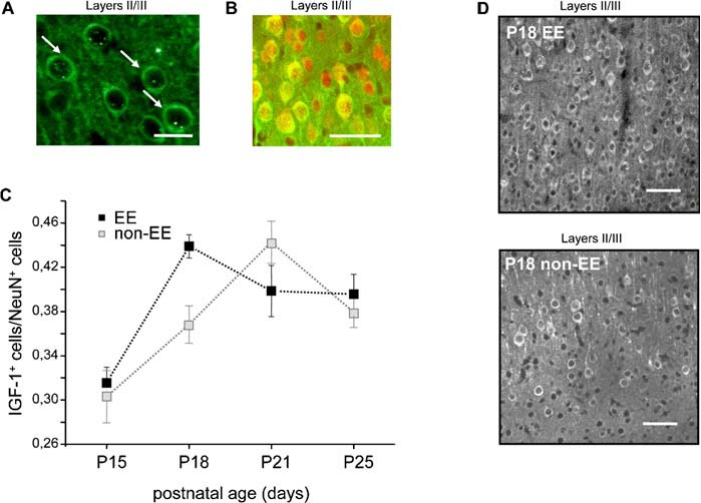
Immunoreactivity for IGF-1 in the developing visual cortex: effects of EE. (A) Typical appearance of IGF-1 positive cells in the developing rat binocular visual cortex Oc1B. Age of the animal, P25, calibration bar 25 µm. (B) Example showing the preponderance of the neuronal phenotype in IGF-1 positive cells in the developing rat binocular visual cortex Oc1B. Age of the animal P18. Staining for IGF-1 green, staining for NeuN (neuronal marker) red, merged image. Calibration bar: 50 µm. (C) Mean number of IGF-1 positive cells in the visual cortex, normalized to the number of neurons (Neu N positive cells) for each developmental age analysed. Black dots are data from EE rats and light grey dots data from non-EE rats. Vertical bars represent SEM. The number of animals analyzed is: for non-EE rats, N = 5 at P15, N = 8 at P18, N = 6 at P21, N = 6 at P25; for EE rats, N = 6 at P15, N = 10 at P18, N = 6 at P21, N = 7 at P25. The normalized number of IGF-1 positive cells increases between P15 and P21 in non-EE rats (Two Way ANOVA, housing (two levels)×age (four levels), factor age significant, p<0,001; *post-hoc* Tukey's test, p<0,05). In EE rats the normalized number of IGF-1 positive cells increases significantly between P15 and P18; the normalized number at P18 in EE rats is significantly increased with respect to non-EE rats (Two Way ANOVA, housing (two levels)×age (four levels), factor age significant, p<0,001, interaction housing×age significant, p = 0,011; *post-hoc* Tukey's test, p<0,05). (D) Example of IGF-1 labelling from fields taken in the layers II/III of the rat visual cortex of one P18 EE and one P18 non-EE rat. It is evident the increase in IGF-1 positive cells caused by EE. Calibration bar: 50 µm.

IGF-1 protein was revealed by means of an immunohistochemical protocol repeatedly used to analyse IGF-1 presence in the central nervous system [23], [21], [43]. Typical appearance of IGF-1 positive cells is shown in Fig. 1A.

At all ages, the great majority of IGF-1 positive cells are neurons (Fig. 1B) both in non-EE and EE rats (Two Way ANOVA, housing×age, factor housing p = 0,16, factor age p<0,001, no significant interaction age×housing). The fraction of IGF-1 positive cells also labelled for NeuN (IGF-1 and NeuN double labelled cells/IGF-1 labelled cells) is: at P15, EE: 0,74±0,02 N = 5; non-EE: 0,8±0,04 N = 4; at P18, EE: 0,89±0,03 N = 4; non-EE: 0,88±0,02 N = 5; at P21, EE: 0,78±0,03 N = 3; non-EE: 0,79±0,02 N = 4; at P25, EE: 0,79±0,04 N = 3; non-EE: 0,83±0,02 N = 4. We quantified the presence of IGF-1 in the visual cortex at different developmental ages in terms of the number of IGF-1 positive cells normalized to the number of neurons (NeuN positive cells; Fig. 1C). The normalized number of IGF-1 positive cells increases between P15 and P21 in non-EE rats (Two Way ANOVA, housing (two levels)×age (four levels), factor age significant, p<0,001, normalized number of IGF-1 positive cells at P21 in non-EE rats 0,44±0,02 (N = 6) differs from the value at P15, 0,3±0,02 (N = 5) and P18, 0,37±0,02 (N = 8); *post-hoc* Tukey's test, p<0,05). In EE rats the normalized number of IGF-1 positive cells at P18 (0,44±0,01, N = 10) is higher than at P15 (0,32±0,01 (N = 6)) and is increased with respect to P18 non-EE rats (Fig. 1C and D) (Two Way ANOVA, interaction housing age significant, p = 0,011, housing within P18, normalized number of IGF-1 positive cells in P18 EE rats differs from P18 non-EE rats, age within EE, EE P18 differs from EE P15; *post-hoc* Tukey's test, p<0,05). An example of the effect of EE on the number of IGF-1 positive cells at P18 is reported in Fig. 1D. The increase in IGF-1 positive cells caused by EE at P18 is due to the increase in IGF-1 positive neurons: indeed, the number of neurons positive for IGF-1 (cells double labelled for NeuN and IGF-1) normalized to NeuN positive cells is significantly different between EE and non-EE rats at P18 (t-test, p = 0,015).

At no age the number of NeuN positive cells is increased by EE with respect to non-EE animals (Two Way ANOVA, age×housing, factor age not significant, p = 0,345, factor housing not significant, p = 0,457); therefore, the increase in the density of IGF-1 positive neurons caused by EE is due to an increased presence of IGF-1 labelled neuronal cells, not to an increase in the density of neurons.

Thus, EE accelerates the developmental time course of IGF-1 protein levels in the visual cortex.

At P18, a double labelling was also performed for IGF-1 and GAD67 to identify whether the IGF-1 positive cells were inhibitory or excitatory neurons. The density of IGF-1 positive inhibitory interneurons is significantly increased by EE (23%±3,8% in non-EE rats and 37%±2,6% in EE rats, t-test, p = 0,03). Thus, EE affects IGF-1 labelling both for excitatory and inhibitory neurons in the developing visual cortex.

### IGF-1 administration in the visual cortex accelerates visual acuity development

**Figure 2:**
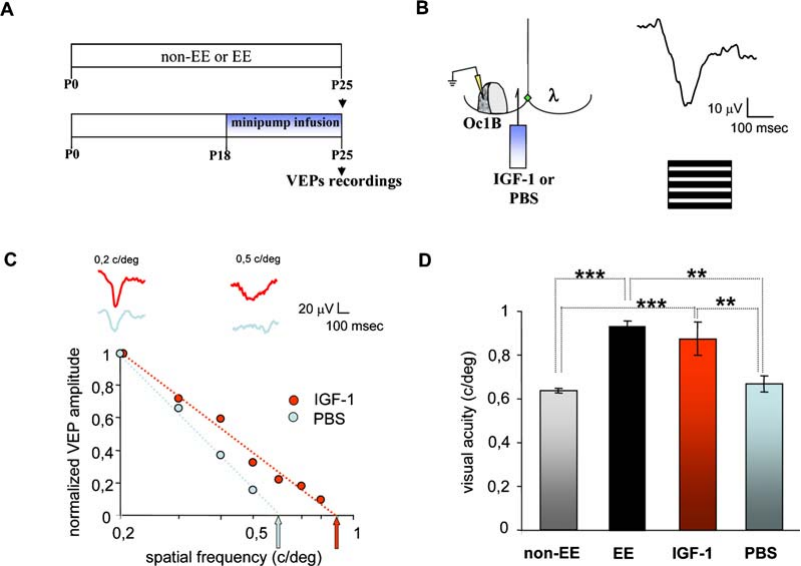
IGF-1 administration in the visual cortex accelerates visual acuity development. (A) Experimental protocol. (B) Left, schematic representation of minipump implant and recording site for Visual Evoked Potentials (VEPs). Right, representative waveform of VEP recorded from Oc1B in response to visual stimulation with gratings sinusoidally modulated in contrast at 1Hz. (C) Example of visual acuity estimate in one IGF-1 (red) and one vehicle (light blue) treated animal. Experimental points are VEP amplitudes normalized to the mean amplitude of VEP at 0,2 c/deg; thick lines are linear fits to the data. Estimated visual acuities (arrows) are taken as the extrapolation to 0 level of the fitting line. Waveforms above the graph are the VEP recordings obtained at 0,2 and 0,5 c/deg for the IGF-1 (red) and the vehicle (PBS) treated animal (light blue). It is evident that at the higher spatial frequency response is obtained only in the IGF-1 treated rat. (D) Summary of visual acuity in all groups. Data are mean visual acuity and vertical bars represent SEM. Visual acuity of non-EE IGF-1 treated animals (IGF-1, 0,9±0,08 c/deg, N = 5) is significantly higher than in non-EE vehicle treated animals (PBS, 0,67±0,03 c/deg, N = 6) or in non-EE untreated animals (non-EE 0,63±0,01 c/deg, N = 7); the latter two do not differ (One Way ANOVA, p<0,001; *post-hoc* Tukey's test, significance level 0,05). The visual acuity in non-EE IGF-1 treated rats do not differ from that in P25 EE rats (EE, 0,93±0,03, N = 4) (One Way ANOVA, *post-hoc* Tukey's test p>0,05). Asterisks denote significant difference (two asterisks, p<0,01, three asterisks p<0,001).

We assessed whether an increase in IGF-1 levels in the visual cortex of non-EE rats from P18 to P25, achieved by infusing exogenous IGF-1 in the visual cortex, mimics EE effects on visual acuity development. IGF-1 (1 µg/µl) was infused by a minipump connected to a cannula implanted 1 mm lateral to lambda [44]–[46]. Visual acuity was assessed by VEP recordings at P25, after a week of IGF-1 treatment (Fig. 2A, B). As control, animals implanted at P18 with PBS filled minipumps and recorded at P25 were used. Two other groups of animals were recorded at P25, animals reared in standard cages (non-EE rats) and animals reared from birth in enriched cages (EE rats), to compare the effects of IGF-1 infusion with those of EE.

The diffusion of IGF-1 to the binocular portion of the primary visual cortex (Oc1B), where VEPs were recorded from, was assessed immunohistochemically at P25 in 4 animals infused with IGF-1 from P18 evaluating the number of IGF-1 positive cells in Oc1B; for comparison, the contralateral cortex, infused with PBS, was used. We found that the density of IGF-1 positive cells (number of IGF-1 positive cells divided by number of neurons (NeuN positive cells) is significantly increased in the IGF-1 treated Oc1B with respect to the contralateral Oc1B (paired t-test, p<0,05) and the number of NeuN positive cells is not increased (paired t-test, p = 0,884). A higher IGF-1 labelling was still visually detectable in visual areas more lateral than Oc1B.

We found that IGF-1 accelerates visual acuity development (Fig. 2); indeed visual acuity of P25 IGF-1 treated animals (0,9±0,08 c/deg, N = 5) is significantly higher than non-EE vehicle treated (0,67±0,03 c/deg, N = 6) or untreated animals (0,63±0,01 c/deg, N = 7) (One Way ANOVA, p<0,001; *post-hoc* Tukey's test, p<0,01 for IGF-1 *vs* vehicle treated rats and vehicle treated *vs* EE rats; p<0,001 for IGF-1 treated *vs* non-EE rats and non-EE *vs* EE rats); the latter two do not differ (One Way ANOVA p<0,001, *post-hoc* Tukey's test p>0,05; Fig. 2D); the effects of IGF-1 treatment are comparable with those produced by EE; visual acuity of P25 IGF-1 treated rats does not differ from that of P25 EE rats (0,93±0,03 c/deg, N = 4, One Way ANOVA p<0,001, *post-hoc* Tukey's test p>0,05; Fig. 2D).

### Block of IGF-1 in the visual cortex prevents the acceleration of visual acuity development in enriched animals

**Figure 3:**
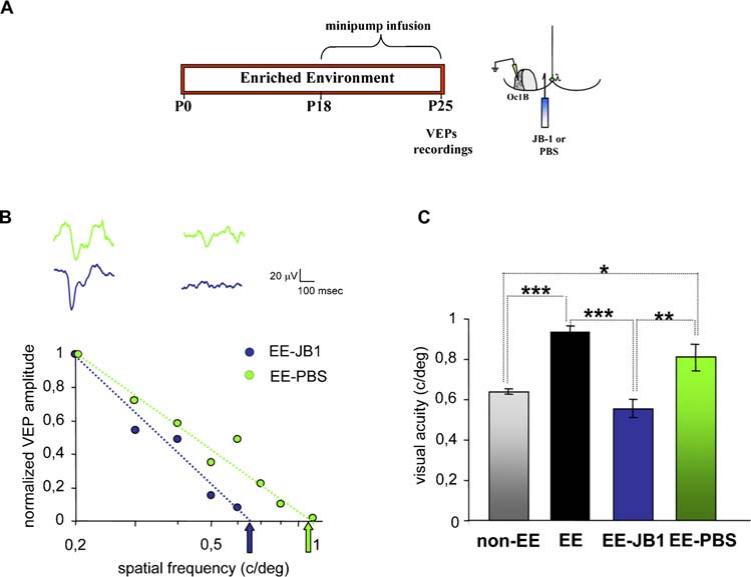
IGF-1 blockade prevents the acceleration of visual acuity development in enriched animals. (A) Experimental protocol and schematic representation of minipump implant and recording site for VEPs. (B) Example of visual acuity estimated in one JB1 treated EE rat (EE-JB1, blue) and one vehicle treated EE animal (EE-PBS, green). Experimental points are normalized VEP amplitudes; thick lines are linear fits to the data. Estimated visual acuities are indicated by arrows. Waveforms above the graph are VEPs recorded in response to visual stimulation with gratings of spatial frequencies 0,2 and 0,5 c/deg for the JB1 treated (blue) and the vehicle treated EE animal (green). It is evident that at the higher spatial frequency a response is obtained only in the vehicle treated EE rat. (C) Summary of mean visual acuity in all JB1 (0,55±0,05 c/deg, N = 5) and PBS treated (0,81±0,07 c/deg, N = 4) P25 EE animals; data for P25 EE and non-EE rats are replotted from Fig. 2 for comparison. Vertical bars represent SEM. Visual acuity of JB1 treated EE animals is significantly lower than in EE animals either treated with vehicle or untreated and does not differ from the visual acuity of P25 non-EE rats (One Way ANOVA, p<0,001; *post-hoc* Tukey's test, significance level 0,05). Asterisks denote significant difference (one asterisk, p<0,05; two asterisks, p<0,01; three asterisks, p<0,001).

To assess if IGF-1 is a crucial factor mediating EE effect on visual acuity development, we performed also the experiment of antagonizing IGF-1 action in EE rats. IGF-1 action was antagonized in the visual cortex from P18 to P25 infusing the IGF-1 receptor antagonist JB1 (Fig. 3A) (concentration in the minipump 10 µg/ml, as in Fernandez et al. [47]). JB1 has been repeatedly used to block IGF-1 [48]–[50] and in particular in the central nervous system [47], [23]. In JB1 treated EE animals we have measured visual acuity at P25 to assess whether antagonizing IGF-1 blocked EE action on visual acuity development. As control, EE animals implanted with PBS containing minipumps were recorded at P25.

To control for possible adverse effects of antagonizing IGF-1 action with JB1 on visual cortical neurons, we have assessed the density of NeuN positive cells at P25 in 5 animals implanted at P18 with a JB1 filled minipump in one cortex and with a PBS filled minipump in the contralateral cortex. We found that neuronal density in the JB1 treated Oc1B (2661±44 NeuN^+^cells/mm^2^) does not differ from that in the contralateral Oc1B (2602±63 NeuN^+^cells/mm^2^, paired t-test p = 0,532); neither the density in the JB1 treated nor that in the PBS treated Oc1B differ from that in Oc1B of untreated of P25 rats (2440±91 NeuN^+^/mm^2^, One Way ANOVA, p = 0,107). The cortical thickness in Oc1B is not affected by JB1 treatment (JB1 treated cortex: 0,915±0,02 mm; PBS treated cortex: 0,87±0,01 mm, paired t-test, p = 0,052). We also recorded non-EE animals implanted with JB1 filled minipumps from P18 to P25 (N = 4, recordings at P25) and from P21 to P28 (N = 4, recordings at P28). We found no difference between visual acuity assessed in these two groups of animals and the visual acuity assessed in non-EE rats of the same age [JB1 treated P25 non-EE, 0,57±0,04 c/deg, untreated P25 non-EE (same data as for Fig. 2) 0,63±0,01 c/deg, p = 0,12, t-test; JB1 treated P28 non-EE rats, 0,75±0,03 c/deg, untreated P28 non-EE-rats (N = 3), 0,79±0,01 c/deg, p = 0,263, t-test)]. Thus, JB1 treatment, at the concentration employed by us, does not seem to have negative effects on the visual cortex.

JB1 treatment blocks EE effects on visual acuity maturation. As shown in Fig. 3, visual acuity of P25 JB1 treated EE animals (0,55±0,05 c/deg, N = 5) is significantly lower than in P25 EE animals either treated with vehicle (0,81±0,07 c/deg, N = 4) or untreated (0,93±0,03 c/deg, N = 4, same data as for Fig. 2D) and does not differ from the visual acuity of P25 non-EE rats (0,63±0,01 c/deg, N = 7, same data as for Fig. 2D) (One Way ANOVA, p<0,001, *post-hoc* Tukey's test, EE untreated *vs* EE JB1 treated rats, p<0,001, EE vehicle treated *vs* EE JB1 treated rats, p<0,01, EE JB1 treated *vs* non-EE rats, p>0,05, EE *vs* EE vehicle treated rats, p>0,05).

Thus, antagonizing IGF-1 action completely prevents EE effects on visual acuity development.

### IGF-1 affects the density of inhibitory synapses and of perineuronal nets in the visual cortex

How could IGF-1 increase mediate EE effects on visual acuity development? One factor which is likely to be relevant for visual acuity development is the intracortical inhibitory tone. The increase of visual acuity is well correlated with a decrease of mean receptive field size of neurons in the primary visual cortex [38] and with the postnatal development of intracortical inhibition [36], [29], [37] which plays a crucial role in shaping visual cortical receptive fields [51], [52]. Dark rearing, which prevents visual acuity development [38], also affects the developmental increase of GABAergic inhibition [53], [37], [54]. BDNF overexpressing mice, which exhibit a precocious development of intracortical inhibition, also show an accelerated development of visual acuity [29]. EE, which accelerates visual acuity development [11], [31] and prevents dark rearing effects on visual acuity [12], also affects the developmental expression of GAD65/67 [11] and prevents dark rearing effects on intracortical inhibition development [12].

**Figure 4:**
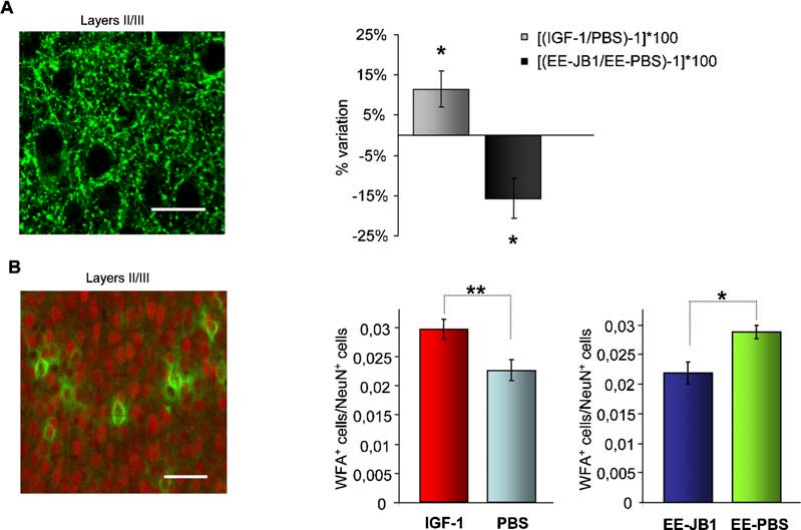
IGF-1 affects intracortical inhibition and perineuronal nets (PNNs) in the developing visual cortex. (A) Left, representative example of GAD65 immunoreactivity in the rat visual cortex at P25. It is evident the punctate nature of the staining around cell bodies (puncta-ring). To quantify GAD65 immunoreactivity in puncta rings, immunofluorescence in puncta ring was normalized to background signal. Calibration bar 20 µm. Right: light bar: percentage variation of GAD65 puncta ring immunoreactivity between the cortex implanted at P18 with a IGF-1 filled minipump and the cortex implanted with a PBS filled minipump in P25 non-EE animals (N = 7). Percentage variation computed as [(GAD65 immunoreactivity in IGF-1 treated/GAD65 immunoreactivity in PBS treated cortex) –1]×100. GAD65 immunoreactivity is significantly higher in the IGF-1 treated than in the PBS treated cortex (paired t-test, p<0,05, one asterisk). Right, dark bar: Percentage variation of GAD65 puncta ring immunoreactivity between the cortex implanted at P18 with a JB1 filled minipump and the cortex implanted with a PBS filled minipump in P25 EE animals (N = 5). Percentage variation computed as [(GAD65 immunoreactivity in JB1 treated/GAD65 immunoreactivity in PBS treated cortex) –1]×100. GAD65 immunoreactivity is significantly lower in the JB1 treated than in the PBS treated cortex (paired t-test, p<0,05, one asterisk). Vertical bars indicate SEM. (B) Left, representative example of WFA staining (green) and NeuN staining (red) merged image in the rat visual cortex at P25. WFA stained PNN completely surround cortical neurons. Calibration bar 50: µm. Right, leftmost: PNN surrounded cells (WFA positive cells/NeuN positive cells) are more numerous in the visual cortex treated from P18 to P25 with IGF-1 than in the contralateral, PBS treated cortex of non-EE animals (N = 5 animals, paired t-test, p<0,01, two asterisks). Right, rightmost: PNN surrounded cells (WFA positive cells/NeuN positive cells) are less numerous in the visual cortex treated from P18 to P25 with JB1 than in the contralateral, PBS treated cortex of EE animals (N = 6 animals, paired t-test, p<0,05, one asterisks). Vertical bars represent SEM.

We have therefore investigated whether the development of GABAergic intracortical inhibition was affected by IGF-1 infusion in the visual cortex assessing the presence of perisomatic inhibitory innervation [29]. Perisomatic innervation mediated by basket interneurons, which constitutes up to 50% of GABAergic interneurons in the visual cortex, is likely an important component of the overall developmental maturation of GABAergic innervation in the primary visual cortex and has been previously used to characterize intracortical inhibition development [29], [12], [55]. Huang et al., [29] found that the development of GABAergic perisomatic inhibition is not completed before the fifth postnatal week in mice. We have quantified the expression of GAD65 in the presynaptic boutons of GABAgergic interneurons around the soma of target neurons (perisomatic puncta rings, [29], [12]) at P25, the age of visual acuity assessment, in non-EE animals (N = 7) implanted at P18 with an IGF-1 filled minipump in one cortex and a PBS filled minipump in the contralateral cortex and in EE animals (N = 5) implanted at P18 with JB1 filled minipump in one cortex and a PBS filled minipump in the contralateral cortex. We have found that GAD65 immunoreactivity in puncta rings was significantly higher in the visual cortex treated with IGF-1 than in the PBS treated cortex (paired t-test, p<0,05, one asterisk) (Fig. 4A, right, light bar). Conversely, it was significantly lower in the visual cortex treated with JB1 than in the PBS treated cortex of EE animals (paired t-test, p<0,05, one asterisk) (Fig. 4A, right, dark bar). Thus, IGF-1 increase could be a mediator of EE effects on visual acuity development via an effect on inhibitory system development. To assess whether this IGF-1 action on inhibitory interneurons was direct or indirect, we determined the presence of IGF-1 receptor on GABAergic interneurons both at P18 and at P25, the beginning and the end of our IGF-1 treatment (see Text S1: Supporting materials and methods). IGF-1 receptor is abundantly expressed in the visual cortex at both ages (Fig. S1). To quantify its presence on GABAergic interneurons we performed a double stain for IGF-1 receptor and GAD67, one of the GABA biosynthetic enzymes (Fig. S2). We found that both at P18 and at P25 the great majority of GAD67 positive interneurons (96% at P18, 76% at P25, no significant difference, Mann-Whitney Rank sum test, p>0,05 ) were also positive for IGF-1 receptor labelling. Thus, a direct effect of IGF-1 on GABAergic interneurons is possible.

The maturation of visual acuity is correlated with the developmental decline of plasticity in the visual cortex [15]. We have recently shown [12] that EE is able to prevent dark rearing effects on the developmental organization into perineuronal nets (PNNs) of Chondroitin Sulphate Proteoglycans (CSPGs), components of the extracellular matrix recently shown to be non permissive factors for visual cortical plasticity [39], [40]. Moreover, EE affects the developmental time course of synaptic plasticity in the visual cortex [11] and counteracts dark rearing effects on the critical period for ocular dominance plasticity [12]. Tropea et al. [28] have shown that IGF-1 is involved in ocular dominance plasticity. We have therefore investigated whether IGF-1 increase in standard animals or IGF-1 blockade in EE animals, around P18 affects PNN development. The density of PNN surrounded neurons increases from P22 to P70 [39]. We examined PNN formation using Wisteria floribunda lectin (WFA) as in [39]; the density of PNN surrounded cells has been determined at P25 in non-EE animals (N = 5) implanted at P18 with an IGF-1 filled minipump in one cortex and a PBS filled minipump in the contralateral cortex and in EE animals (N = 6) implanted at P18 with a JB1 filled minipump in one cortex and a PBS filled minipump in the contralateral cortex. We have found that the density of PNN surrounded cells (number of WFA positive cells normalized to number of NeuN positive cells) was significantly higher in the IGF-1 treated cortex than in the contralateral cortex (paired t-test; p<0,01 two asterisks) (Fig. 4B, right, leftmost), while it was significantly lower in the visual cortex treated with JB1 than in the PBS treated cortex (paired t-test, p<0,05, one asterisk) (Fig. 4B, right, rightmost). Thus, IGF-1 is likely a mediator of EE effects on PNN development and, through it, on visual cortex experience dependent plasticity.

To assess whether this IGF-1 action on PNN surrounded interneurons was direct or indirect, we determined the presence of IGF-1 receptor on PNN surrounded interneurons both at P18 and at P25 (see Text S1: Supporting materials and methods). We performed a double stain for IGF-1 receptor and WFA, to label PNNs (Fig. S3). We found that both at P18 and at P25 a large proportion of WFA positive interneurons (64% at P18, 50% at P25, p = 0,03, Mann-Whitney Rank sum test) were also positive for IGF-1 receptor labelling. Thus, a direct effect of IGF-1 on PNN surrounded interneurons is possible.

## Discussion

We have found that IGF-1 is involved in mediating the effects of experience on postnatal cortical development and in particular is a key factor mediating EE effects on visual acuity development. We suggest that IGF-1 could act via an effect on the development of the intracortical inhibitory circuitry and of PNNs.

IGF-1 expression in the developing brain is generally transient and different regions show different time courses of IGF-1 developmental expression; in each system, IGF-1 expression appears during relatively late stages of their development, at a time of maturation of dendrites and synapse formation [32]. We found that IGF-1 protein expression in the visual cortex increases between P15 and P21, a period of active synaptogenesis in all cortical layers [56] and which corresponds to the beginning of the critical period for experience-dependent remodelling of visual connections in the rat [38]. This is consistent also with the role for IGF-1 in experience dependent visual cortical plasticity, as recently suggested by Tropea et al., [28].

The first indication that IGF-1 might be involved in the effects produced by EE on the developing visual cortex is that IGF-1 expression in the visual cortex is affected by EE. In particular, IGF-1 immunoreactivity at P18 is higher in rats exposed to EE (EE rats) than in non-EE rats.

At all ages, the great majority of IGF-1 positive cells are neurons. The number of NeuN positive cells is not increased by EE; therefore, the increase in the density of IGF-1 positive neurons in EE animals is due to an increased presence of IGF-1 labelled neuronal cells, not to an increase in the density of neurons. Also the density of IGF-1 positive inhibitory interneurons in the visual cortex is significantly increased by EE at P18. Thus, we show for the first time that IGF-1 presence in the developing visual cortex is sensitive to the experience provided by EE and that both excitatory neurons and inhibitory interneurons could be potentially affected by IGF-1 increase.

During development, IGF-1 expression in neurons is well documented [32], [33]. The effects of EE on IGF-1 protein levels in the visual cortex could therefore be attributed to an increased IGF-1 mRNA expression in the visual cortex. However, we cannot exclude a contribution to the increased IGF-1 immunoreactivity found in P18 EE rats from an increase in circulating IGF-1, a decrease in IGF-1 binding protein [28] or an increase in IGF-1 receptors on visual cortical neurons [27].

The observations that exogenous IGF-1 supply from P18 mimics, while block of IGF-1 action from P18 prevents, EE effects on visual acuity development is, in our opinion, a very strong evidence that IGF-1 increase is both sufficient and necessary for EE to affect visual acuity development. The effects of infusing either IGF-1 or IGF-1 blocker JB1 in the visual cortex cannot be attributed to aspecific effects of infusion since they are absent in vehicle infused animals.

Visual acuity is a sensitive index of visual cortical development [29], [30]; thus, our observations suggest that IGF-1 is an important factor in mediating EE effects on the development of the visual cortex. At the same time, they show for the first time that IGF-1 is able to accelerate visual acuity development.

How could IGF-1 affect visual acuity development? One hypothesis stems from the known effects of IGF-1 in increasing neuronal activity [23]. The enhanced presence of IGF-1 in EE rat visual cortex might act on neurons bearing IGF-1 receptors, determining an increase in spontaneous or evoked neural activity, and in the production of activity-dependent factors, such as neurotrophins [13], or in the activation of activity-dependent pathways, such as ERK/CREB, important for visual cortical development and plasticity [46], [16]. Consistently with this possibility, Cancedda et al. [11] found that in non-EE mice the peak of CRE mediated gene expression in the visual cortex is at P25, while in EE mice it is anticipated at P20, which is in good agreement with the increase of IGF-1 immunoreactivity we found at P18 in EE rats. It would be important to assess whether IGF-1 administration activates the ERK/CREB pathway in the visual cortex and in which neuronal types.

We have shown for the first time that IGF-1 labelling is present in inhibitory interneurons in the developing visual cortex and that the density of IGF-1 positive interneurons is increased by EE. This suggests that another, non alternative, explanation for IGF-1 effects on visual acuity development could be an action of IGF-1 on inhibitory interneurons. The observation that IGF-1 affects GAD65 immunoreactivity in puncta-rings demonstrates that inhibitory interneurons respond to IGF-1 with a GAD65 increase in their synaptic terminals, an effect possibly mediated by an increase in BDNF expression, which is known to be caused by IGF-1 in the adult [23], [25] and which affects intracortical inhibitory system development in the visual cortex [29]. The result that GABAergic interneurons in the visual cortex express IGF-1 receptor during development suggests that IGF-1 can also act directly on inhibitory interneuron development. The nature of this action, whether it is directed on neuronal activity or/and on the expression of synaptic proteins and GABA biosynthetic enzymes remains to be determined. Since inhibitory interneuron development has been suggested to contribute to visual acuity development [29], [12] we suggest that IGF-1 action on inhibitory interneuron development is a possible mediator of EE effects on visual acuity development, although a contribution from an IGF-1 action on excitatory neuron development cannot be excluded.

We have also shown for the first time that IGF-1 affects PNN development. The great majority of PNN surrounded neurons are inhibitory interneurons [39], [57]. The precocious development of PNNs caused by IGF-1 could contribute to inhibitory interneuron development, contributing to visual acuity development.

The action of IGF-1 on PNNs and inhibitory interneurons suggests that IGF-1 is also a mediator of EE effects on visual cortical plasticity shown in [11] and [12]. This would be in agreement with the involvement of IGF-1 in ocular dominance plasticity in response to monocular deprivation [28].

## Materials and Methods

### Subjects and mating protocol

All experiments were performed on rats in accordance with the Italian Ministry of Public Health guidelines for care and use of laboratory animals.

Long Evans hooded rats lived in an animal house with a temperature of 21°C, 12 h/12 h light/dark cycle, and food and water available ad libitum. For both housing conditions, matings were made inside the cage. After birth all the litters were housed with their mother until the date of experiment.

#### Rearing environments

Enriched environment (EE) consisted of large wire netting cages (60×50×80 cm) with three floors containing several foodhoppers, two running wheels (one bigger for adults, the other for post-weaning pups) to allow physical activity, and differently shaped objects (tunnels, shelters, stairs) that were completely substituted with others once a week. Every cage housed at least 4–5 females and their pups. Cages for standard environment (non-EE) were standard laboratory cages (30×40×20 cm) housing one dam with her pups as established by the Italian law for the care of laboratory animals.

### IGF-1 and JB1 intracortical administration

Drugs (IGF-1 or JB1) were infused with osmotic minipumps (model 1007D; 0,5 µl/h; Alzet, Palo Alto, CA) starting at P18. Minipumps were connected via PVC tubing to a stainless steel 30-gauge cannula implanted 1 mm lateral to lambda of the left visual cortex [44], [45]. IGF-1 (IBT; 1 µg/µl) was infused in the visual cortex of non-EE rats (N = 12), while JB1 (Bachem AG; 10 µg/ml) was infused in the visual cortex of EE rats (N = 11), and non-EE rats (N = 5). As control, PBS was infused. All treatments were made for one week between P18 and P25. We also recorded non-EE animals implanted with JB1 filled minipumps from P18 to P25 (N = 4, recordings at P25) and from P21 to P28 (N = 4, recordings at P28).

### Electrophysiological assessment of visual acuity with Visual Evoked Potentials

A total of 39 rats [animals non enriched (non-EE): N = 7; enriched (EE): N = 4; non-EE treated with IGF-1 (IGF-1): N = 5; non-EE treated with vehicle (PBS): N = 6; EE treated with JB1(EE-JB1): N = 5; EE treated with vehicle (EE-PBS): N = 4; non-EE treated with JB1: N = 8] was used for electrophysiology. Rats were anesthetized with an intraperitoneal injection of 20% urethane (0,7 ml/hg; Sigma, St. Louis, MO) and mounted on a stereotaxic apparatus allowing full viewing of the visual stimulus. Additional doses of urethane (0,03–0,05 ml/hg) were used to keep anaesthesia level stable throughout the experiment. During electrophysiology, the body temperature of rats was monitored with a rectal probe and maintained at 37.0°C with a heating pad. Visual stimuli were horizontal sinusoidal gratings of different spatial frequency and contrast generated by a VSG2/2 card (Cambridge Research System, Cheshire, UK) and presented on a computer display (mean luminance = 25 candles/m^2^; area, 24×26 cm) placed 20 cm in front of the animal. Recordings were always made in blind in relation to the animal's rearing condition to avoid subjective judgements of the experimenter.

#### Visual Evoked Potentials (VEPs)

VEPs were recorded as in [46]. Briefly, a large portion of the skull overlying the binocular visual cortex was drilled and removed taking away the dura. A glass micropipette (2-2,5 ΜΩ) was inserted into the binocular primary visual cortex (Oc1B; [58]) in correspondence of the vertical meridian representation and advanced 100 or 450 µm within the cortex. At these depths, VEPs had their maximal amplitude. Electrical signals were amplified, bandpass filtered (0,1–120 Hz), and averaged (at least sixty events in blocks of ten events each) in synchrony with the stimulus contrast reversal. Transient VEPs in response to abrupt contrast reversal (0,5–1 Hz) were evaluated in the time domain by measuring the peak-to-baseline amplitude and peak latency of the major component. VEPs in response to a blank field were also frequently recorded to have an estimate of the noise. For each animal, VEP amplitude was plotted as a function of log spatial frequency and visual acuity was determined by linearly extrapolating VEP amplitude to 0 V.

### Immunohistochemistry

A total of 80 (EE: N = 35; non-EE: N = 45) Long Evans hooded rats aged between P15 and P25 were employed (P15, EE: N = 6, non-EE: N = 5; P18, EE: N = 10, non-EE: N = 12; P21, EE: N = 6, non-EE: N = 6; P25, EE: N = 13; non-EE: N = 22). Animals were deeply anesthetized with chloral hydrate and perfused transcardially with PBS 1×followed by fixative (4% paraformaldehyde, 0.1 M sodium phosphate, pH 7.4; PB). Brains were removed, post-fixed in the same fixative at 4°C, cryoprotected by immersion in 30% sucrose with 0,01% sodium azide solution in PB at 4°C and frozen by isopentane. 35 µm coronal sections were cut on a microtome and processed for immunohistochemistry. Free floating sections were incubated for 1–2 hours in a blocking solution (containing 10% BSA, 0,3% Triton X-100 in PBS or 3% BSA in PBS for WFA staining) followed by incubation with the appropriate antibodies.

For IGF-1 we used rabbit polyclonal anti-IGF-1 antibody (1∶500 in 1% BSA, 0,2% Triton; antibody kindly provided by I. Torres-Aleman) revealed with biotinylated secondary antibody goat anti-rabbit IgG (1∶200, Vector Laboratories, Burlingame, CA) followed by fluorescein-conjugated extravidin (1∶300, Sigma). For NeuN we used Chemicon, MAB377, (1∶500, in 1% BSA, 0,2% Triton) revealed with Alexa 568 (Molecular Probes, 1∶400). For GAD67 we used mouse anti GAD67 (Chemicon, MAB5406, 1∶1000 in 1% BSA, 0,3% Triton) revealed with Alexa 568 (Molecular Probes, 1∶400). For WFA staining sections were incubated overnight at 4°C in a solution of biotinylated Wisteria floribunda lectin (WFA) (1∶100, Vector). WFA was stained with 1h incubation in fluorescein-conjugated extravidin (1∶300, Sigma). For GAD65 we used monoclonal antibody anti GAD65 (Chemicon, MAB351, 1∶500, in 1% BSA, 0,2% Triton) revealed with biotinylated secondary antibody goat anti-mouse IgG (1∶200, Vector Laboratories, Burlingame, CA) followed by incubation in fluorescein-conjugated extravidin (1∶300, Sigma). Sections were then mounted on slides with Vectashield.

### IGF-1 immunoreactivity analysis

At all ages, images were acquired with a confocal Olympus microscope at 40×magnification, (N.A. = 0,85, field 353×353 µm acquired at 1024×1024 pixels) to analyze the colocalization of antigens and at 20×(N.A. = 0,7, field 707×707 µm acquired at 1024×1024 pixels) to analyze the number of IGF-1 and NeuN positive cells in sections double labelled for IGF-1 and NeuN. To compare different specimens, the parameters of acquisition were optimized at the start and then held constant throughout image acquisition. The collected images from Oc1B cortical fields were imported to the image analysis system MetaMorph. For each animal, at least three Oc1B sections were analyzed. At each age, counts were done on the entire thickness of Oc1B. The number of IGF-1 positive cells was normalized to the number of NeuN positive cells both in EE and non-EE animals. The number of NeuN positive cells was also compared between EE and non-EE rats for each age. To identify whether the IGF-1 positive cells were neurons, the number of double labelled IGF-1 and NeuN positive cells was counted and, for each age and housing condition, was normalized to the total number of IGF-1 positive cells. To identify the proportion of IGF-1 positive cells which are neurons the number of double labelled IGF-1 and NeuN positive cells was counted and normalized to the total number of NeuN positive cells at P18. To identify whether IGF-1 positive cells were excitatory neurons or inhibitory interneurons and to assess whether EE affected the number of IGF-1 positive inhibitory interneurons, double labelling for IGF-1 and GAD 67 was performed at P18 in EE (N = 4) and non-EE animals (N = 3). For each animal at least 3 Oc1B sections were analyzed; acquisition were done at 40×magnification, zoom 1×(N.A. = 0,85, field 353×353 µm acquired at 1024×1024 pixels). The number of double labelled cells was counted on the entire Oc1B thickness. All image acquisition and analysis were carried out in blind.

### WFA quantification

Images were acquired at 20×(N.A. = 0,7, field 707×707 µm acquired at 1024×1024 pixels). To compare different specimens, the parameters of acquisition were optimized at the start and then held constant throughout image acquisition. For each animal at least 6 Oc1B sections were analyzed (three for IGF-1 or JB1 treated cortex and three for PBS contralateral cortex). The collected images from Oc1B cortical fields were imported to the image analysis system MetaMorph. Counts were done on the entire thickness of Oc1B (mosaic of three 20×images) and the ratio WFA-positive cells/NeuN positive cells was calculated. All images acquisition and analysis were carried out in blind.

### GAD65 puncta rings quantification

Images were acquired at 60×(N.A. = 1,40, field 105×105 µm acquired at 512×512 pixels). Settings for laser intensity, gain, offset and pinhole were optimized initially and held constant through the study. During image collection, confocal settings were regulated so that the full range of pixel intensities (0-255) was used, with very little saturation at either end of intensity range. For each animal at least six sections (three from IGF-1 or JB1 treated cortex and three from PBS contralateral cortex) were analyzed. For each section, we imaged six field taken from layer II/III of the primary visual cortex. In each field, a stack of ten GAD65 optical sections separated by 1um was collected at the top face of the tissue section. The image within each stack with the highest average pixel intensity was selected for the quantitative analysis of GAD65 immunoreactivity [59], [60]. Perisomatic GAD65 signals (“puncta-ring”) from at least three target neurons were outlined for each image and GAD65 signal intensity was calculated (Methamorph). For each neuron, signal intensity were divided by the background labelling in the cell soma. A total sample of 40–50 neurons were analyzed for each cortex. All images acquisition and analysis were carried out in blind.

## Supporting Information

Text S1 Supporting materials and methods
(0.02 MB DOC)

Figure S1 Representative example of staining for IGF-1 receptor (IGF-1R) in the visual cortex of a P18 and a P25 rat. Microphotographs from layers II-III. Calibration bar: 50 μm. It is evident that IGF-1 receptor is abundantly expressed in the visual cortex during this developmental period.
(10.19 MB TIF)

Figure S2 Representative example of double staining for IGF-1 receptor (IGF-1R) (green) and GAD67 (red) in the visual cortex of a P18 and a P25 rat. The merged images show that at both ages the great majority of GAD67 positive neurons also express IGF-1 receptor (yellow labelling). Microphotographs from layers II-III. Calibration bar: 50 μm.
(10.19 MB TIF)

Figure S3 Representative example of double staining for IGF-1 receptor (IGF-1R) (red) and WFA (green) in the visual cortex of a P18 and a P25 rat. The merged images show that at both ages a large proportion of WFA positive neurons also express IGF-1 receptor. Microphotographs from layers II-III. Calibration bar: 25 μm.
(10.19 MB TIF)
