# Supplementary material for: Insulin-Like Growth Factor 1 (IGF-1) Mediates the Effects of Enriched Environment (EE) on Visual Cortical Development
Source: PLoS One. 2007 May 30;2(5):e475. doi: 10.1371/journal.pone.0000475 (PMC1871611; doi:10.1371/journal.pone.0000475)
Supplement: Text S1 — Supporting materials and methods (0.02 MB DOC) [file pone.0000475.s001.doc]

**Supporting Information**

### **Double labelling IGF-1 receptor and GAD67 or IGF-1 receptor and WFA**

To assess the presence of IGF-1 receptor on inhibitory interneurons and on PNN surrounded neurons in the time window of our treatment, double labelling for IGF-1 receptor and either GAD67 or WFA was performed in non-EE animals at P18 (N=4) and at P25 (N=4). We used polyclonal anti-IGF-1R antibody (C-20, sc-713, Santa Cruz, 1:50 in 1% BSA, 0,2% Triton) revealed with Alexa 488 (Molecular Probes, 1:400) for IGF-1R and GAD67 double staining or with Alexa 568 (Molecular Probes, 1:400) for IGF-1R and WFA double staining. Sections were then mounted on slides with Vectashield. For each animal at least 3 Oc1B sections were analysed; acquisition were done at 40 X magnification, zoom 1 X (N.A.=0,85, field 353 x 353 μm acquired at 1024x1024 pixels). The collected images from Oc1B cortical fields were imported to the image analysis system MetaMorph. The number of double labelled cells was counted on the entire Oc1B thickness. All image acquisition and analysis were carried out in blind.
