# Supplementary figures and images for: Insulin-Like Growth Factor 1 (IGF-1) Mediates the Effects of Enriched Environment (EE) on Visual Cortical Development
Source: PLoS One. 2007 May 30;2(5):e475. doi: 10.1371/journal.pone.0000475 (PMC1871611; doi:10.1371/journal.pone.0000475)

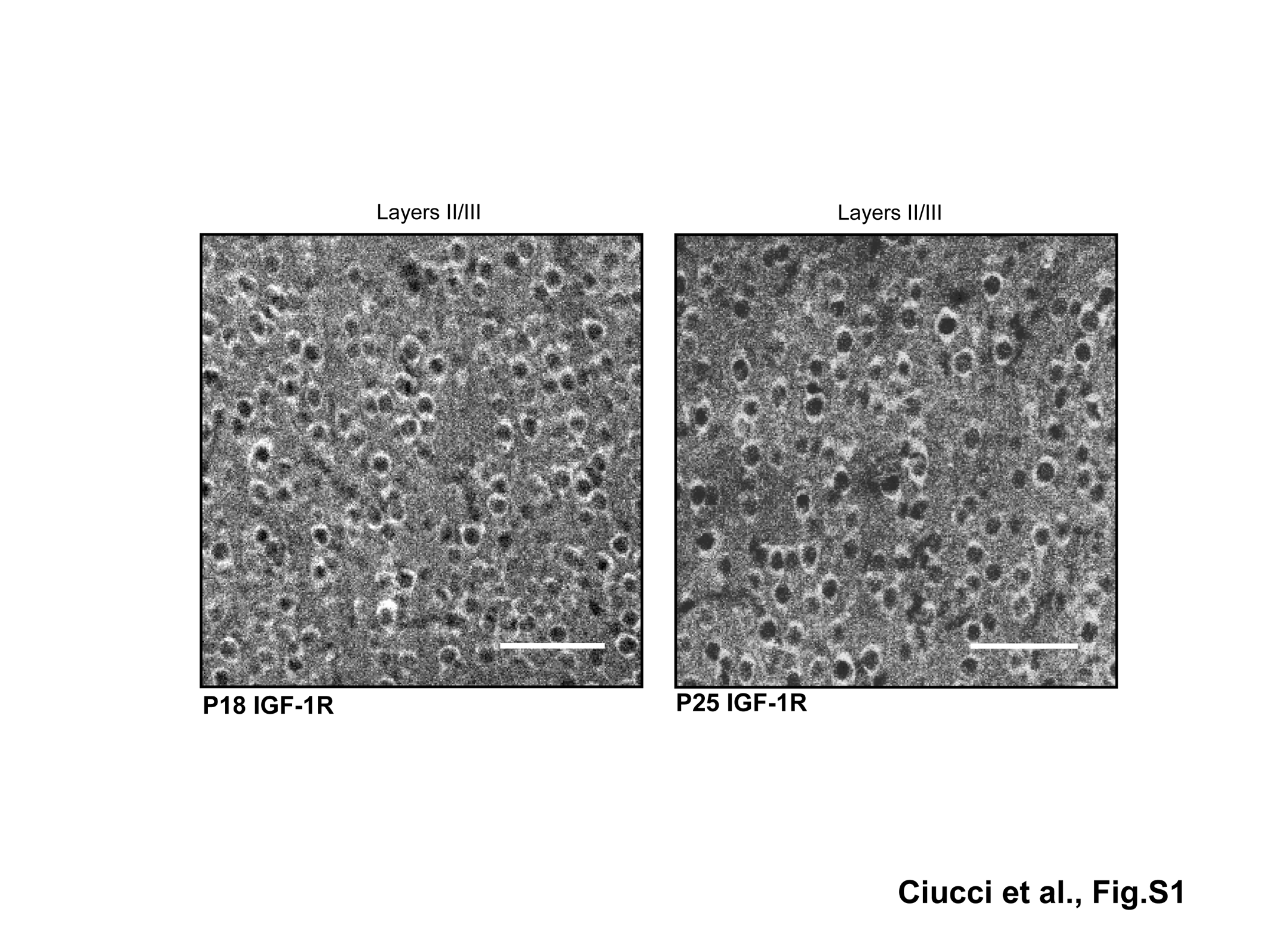

Supplement: Figure S1 — Representative example of staining for IGF-1 receptor (IGF-1R) in the visual cortex of a P18 and a P25 rat. Microphotographs from layers II-III. Calibration bar: 50 μm. It is evident that IGF-1 receptor is abundantly expressed in the visual cortex during this developmental period. (10.19 MB TIF) [file pone.0000475.s002.tif]

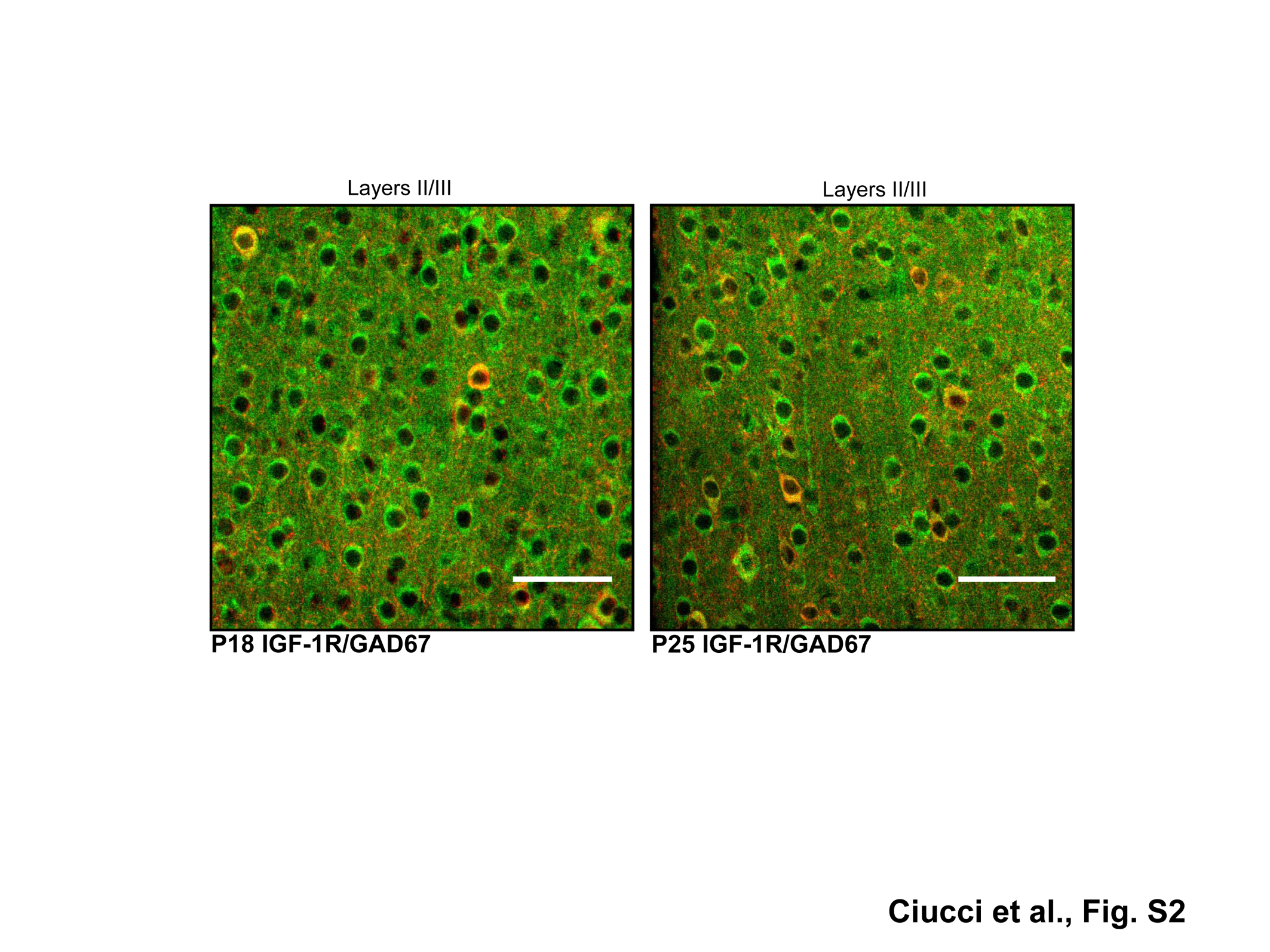

Supplement: Figure S2 — Representative example of double staining for IGF-1 receptor (IGF-1R) (green) and GAD67 (red) in the visual cortex of a P18 and a P25 rat. The merged images show that at both ages the great majority of GAD67 positive neurons also express IGF-1 receptor (yellow labelling). Microphotographs from layers II-III. Calibration bar: 50 μm. (10.19 MB TIF) [file pone.0000475.s003.tif]

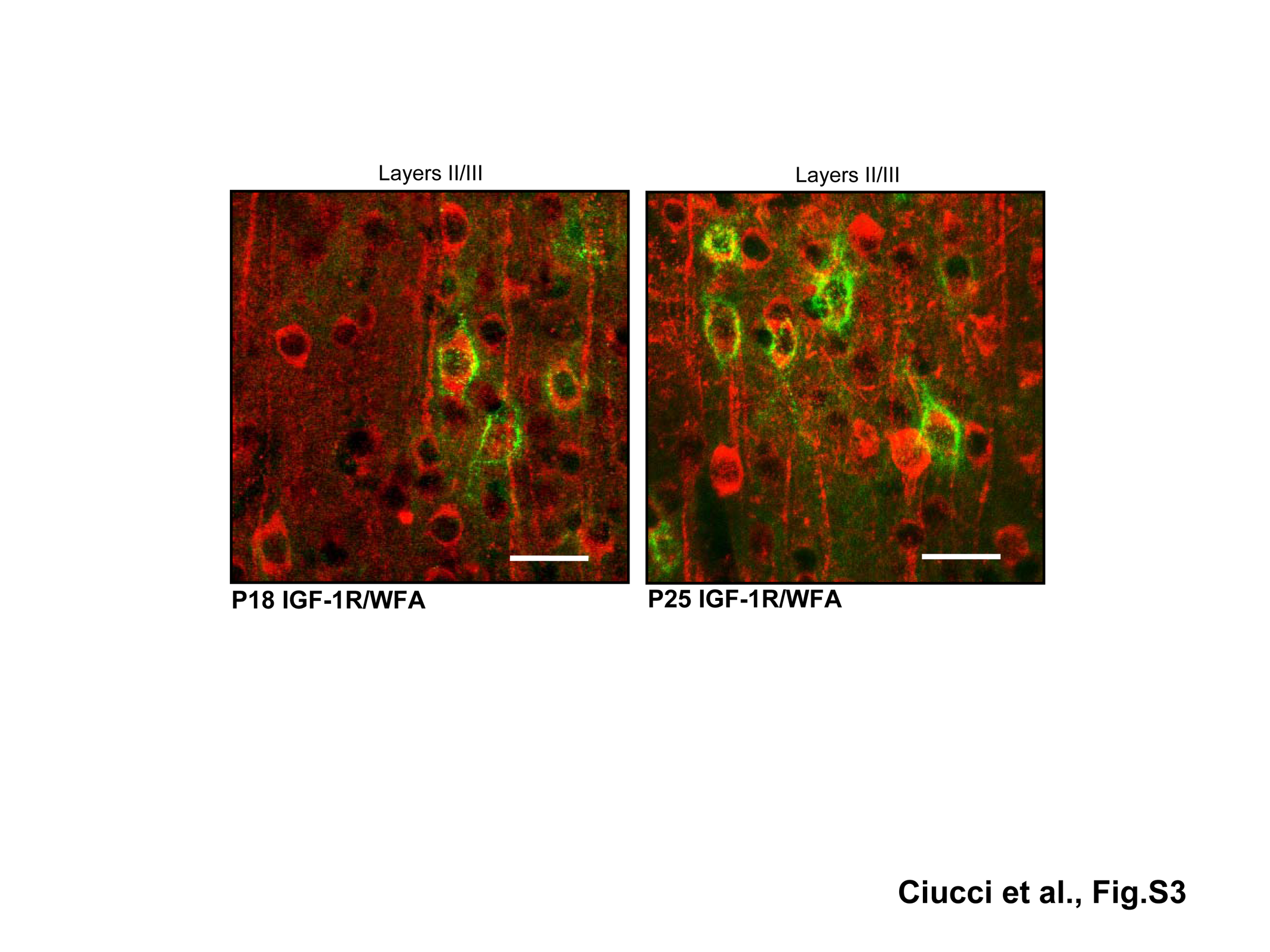

Supplement: Figure S3 — Representative example of double staining for IGF-1 receptor (IGF-1R) (red) and WFA (green) in the visual cortex of a P18 and a P25 rat. The merged images show that at both ages a large proportion of WFA positive neurons also express IGF-1 receptor. Microphotographs from layers II-III. Calibration bar: 25 μm. (10.19 MB TIF) [file pone.0000475.s004.tif]
